# Integrating Explainable Machine Learning in Clinical Decision Support Systems: Study Involving a Modified Design Thinking Approach

**DOI:** 10.2196/50475

**Published:** 2024-04-16

**Authors:** Michael Shulha, Jordan Hovdebo, Vinita D’Souza, Francis Thibault, Rola Harmouche

**Affiliations:** 1 Lady Davis Institute for Medical Research Jewish General Hospital Centre intégré universitaire de santé et de services sociaux (CIUSSS) du Centre-Ouest-de-l'Île-de-Montréal Montreal, QC Canada; 2 Department of Family Medicine McGill University Montreal, QC Canada; 3 National Research Council of Canada Winnipeg, MB Canada; 4 National Research Council of Canada Boucherville, QC Canada

**Keywords:** explainable machine learning, XML, design thinking approach, NASSS framework, clinical decision support, clinician engagement, clinician-facing interface, clinician trust in machine learning, COVID-19, chest x-ray, severity prediction

## Abstract

**Background:**

Though there has been considerable effort to implement machine learning (ML) methods for health care, clinical implementation has lagged. Incorporating explainable machine learning (XML) methods through the development of a decision support tool using a design thinking approach is expected to lead to greater uptake of such tools.

**Objective:**

This work aimed to explore how constant engagement of clinician end users can address the lack of adoption of ML tools in clinical contexts due to their lack of transparency and address challenges related to presenting explainability in a decision support interface.

**Methods:**

We used a design thinking approach augmented with additional theoretical frameworks to provide more robust approaches to different phases of design. In particular, in the problem definition phase, we incorporated the nonadoption, abandonment, scale-up, spread, and sustainability of technology in health care (NASSS) framework to assess these aspects in a health care network. This process helped focus on the development of a prognostic tool that predicted the likelihood of admission to an intensive care ward based on disease severity in chest x-ray images. In the ideate, prototype, and test phases, we incorporated a metric framework to assess physician trust in artificial intelligence (AI) tools. This allowed us to compare physicians’ assessments of the domain representation, action ability, and consistency of the tool.

**Results:**

Physicians found the design of the prototype elegant, and domain appropriate representation of data was displayed in the tool. They appreciated the simplified explainability overlay, which only displayed the most predictive patches that cumulatively explained 90% of the final admission risk score. Finally, in terms of consistency, physicians unanimously appreciated the capacity to compare multiple x-ray images in the same view. They also appreciated the ability to toggle the explainability overlay so that both options made it easier for them to assess how consistently the tool was identifying elements of the x-ray image they felt would contribute to overall disease severity.

**Conclusions:**

The adopted approach is situated in an evolving space concerned with incorporating XML or AI technologies into health care software. We addressed the alignment of AI as it relates to clinician trust, describing an approach to wire framing and prototyping, which incorporates the use of a theoretical framework for trust in the design process itself. Moreover, we proposed that alignment of AI is dependent upon integration of end users throughout the larger design process. Our work shows the importance and value of engaging end users prior to tool development. We believe that the described approach is a unique and valuable contribution that outlines a direction for ML experts, user experience designers, and clinician end users on how to collaborate in the creation of trustworthy and usable XML-based clinical decision support tools.

## Introduction

Though much research has been published on the applications of machine learning (ML) in clinical contexts, few studies have proceeded to deployment for patient care [1]. Barriers to adoption in health care include data quality; data bias; and lack of proper validation, reproducibility, and transparency [2]. Particularly with respect to transparency, black box models, which are increasingly used for prediction tasks in clinical contexts, do not provide the rationale behind the prediction in order to justify a clinical decision [3,4]. In fact, studies found that “physicians need to understand artificial intelligence (AI) methods and systems sufficiently to be able to trust an algorithm’s predictions—or know how to assess the trustworthiness and value of an algorithm—as a foundation for clinical recommendations” [5].

Explainable machine learning (XML) is a field focused on developing techniques to help end users understand the predictions made by complex models [6]. Indeed, we followed Rudin [7] in adopting the definition of XML as the use of additional post-hoc models to explain a primary black-box model. Such black-box models are in contrast to interpretable models. This includes information concerning the underlying data and performance of the model [8]. However, the effectiveness of various approaches for explainability are dependent upon well-designed and highly usable user interfaces [9], and Abdul et al [10] pointed out that much of the work within the domains of AI and ML has not focused on usability or practical interpretability. Indeed, as Liao et al [8] discussed, current work provides limited guidance on actualizing guidelines in user interfaces.

As discussed by Schwartz et al [11], clinician involvement in the design of ML clinical decision support has primarily been used to validate the clinical accuracy of underlying models developed by the researchers. A recent review by Chen et al [12] of explainable AI and ML medical imaging design found no evidence of end-user clinical involvement in the design of explainability models and a highly limited number of articles that documented an empirical assessment of explainability claims with end users. These findings mirror our previous unpublished work that looked at the broader state of XML in clinical decision support and the same low engagement of end users in the empirical assessment of XML decision support applications.

Our study used a design thinking [13,14] approach to explore how constant engagement of clinician end users could provide insights on how to improve the alignment of XML decision support to actual end-user needs and address challenges related to presenting explainability in a decision support interface. To this end, we identified a relevant ML decision support tool targeted toward COVID-19 via clinician focus groups. We then developed a clinician-facing interface for the quantification of COVID-19 severity from chest x-ray images with XML. We tested the resulting prototype via structured interviews with clinicians to verify the domain-appropriate representation, potential actionability, and consistency of the tool.

## Methods

### Ethical Considerations

Ethical approval for this study was granted by the Centre intégré universitaire de santé et de services sociaux (CIUSSS) of West Central Montreal psychosocial research ethics committee (Project 2022-2838) and by the NRC research ethics board (Project 2021-101). Informed consent was received from all participants. In all analysis and research documents, participant-identifying data were replaced by a code. No compensation was offered to any participants of the study.

### Design Thinking Approach

We chose a design thinking approach to optimize clinician involvement in the creation of an XML-based clinical decision support system (CDSS). Design thinking is a process for solving complex problems that emphasizes iteration and rapid prototyping to maximize end-user involvement in generating a usable solution. Stanford University Design School describes 5 key phases of the design thinking approach, namely, *empathize*, *define*, *ideate*, *prototype*, and *test*. Table 1 provides an overview of the work presented in this manuscript according to design thinking phases. For each phase, we define the objective, associated research activities, end-user involvement, and supplementary theoretical frameworks used to add robustness to our work.

To simplify the structure of the paper, we have chosen to report the majority of research activities conducted in the empathize, define, and ideate phases in the Methods section. The Results section is primarily focused on the outputs of the prototype and test phases.

**Table 1:** Overview of the design thinking phases and research activities conducted during each phase.

Phase	Empathize	Define	Ideate	Prototype	Test
Phase objective	Consult experts to better understand the design challenge, and engage and empathize to understand motivations and experiences	Analyze and synthesize observations to identify and define core problems	Brainstorm approaches to achieve solutions	Production of a scaled-down version to iterate different solution ideas with users	Testing of the best solutions
Research activities	Rapid review, focus groups, and scoping review	Data synthesis, analysis, and decision on tool function	Iterative design of the tool user experience; Identification of an appropriate explainability approach	Paper prototype testing; Implementation and testing of different explainability approaches; Development of an interactive working prototype	Software prototype testing; Analysis of results
Physician end-user involvement	Share opinions and experiences in focus groups	Select the potentially most useful tool; Prioritize features	Consult on early design concepts	Provide iterative feedback on paper prototypes	Formally assess a working prototype
Theoretical frameworks used in analysis/design	N/A^a^	NASSS^b^ framework [15]	Framework for clinician trust in machine learning [4]	Framework for clinician trust in machine learning [4]	Framework for clinician trust in machine learning [4]

^a^N/A: not applicable.

^b^NASSS: nonadoption, abandonment, scale-up, spread, and sustainability of technology in health care.

### Empathize Phase

The objective of the empathize phase was to better understand the motivation and experiences of potential end users and to consult with experts on the problem in question. In this phase, we conducted three key research activities: (1) a rapid review to identify clinical use cases for ML or AI that could benefit from explainability and be useful to clinicians in the context of the COVID-19 pandemic; (2) focus groups with physicians designed to review the output of the rapid review and to elicit data that would help the team better understand the scope and nature of a tool that would be most useful to physicians in an integrated health care network, responding to the COVID-19 pandemic; and (3) a scoping review to better understand the existing XML CDSS in health care, the associated design frameworks used for explainability, and the research methods used to study end-user perceptions.

During our rapid review, we searched Scopus, the World Health Organization (WHO) COVID-19 publication database, and the Dialog Proquest COVID-19 database to identify systematic reviews, literature reviews, or surveys of AI or ML technologies used to support clinicians in a pandemic (COVID-19). We identified 65 review articles, of which the 7 most pertinent were used to separate the cited papers within the reviews into 8 broad categories of applications. We selected 4 of these categories to present to stakeholders as candidate applications based on assessment of their clinical need, applicability to hospital settings, relevance to our current research field, interest to clinicians, and feasibility in the chosen clinical setting. The selected categories were large-scale COVID-19 screening; detection, diagnosis, or prognosis of COVID-19; predicting recovery, mortality, or severity of COVID-19 patients; and hospital resource management.

After performing a nonexhaustive scan of additional publications that fit these categories, we retained a total of 37 articles that were peer reviewed and that described ML implementations considering the following criteria: techniques where explainability would be beneficial and associated data or codes were available for implementation. These articles were explored to further select 3 themes, each with its own clinical use case for an ML application, which cross-cut the previously described categories. The first theme was screening. It involved algorithms for tools that may help with COVID-19 screening by predicting the risk of COVID-19 in undiagnosed patients through the analysis of text-based telehealth notes or triage notes in the emergency room. The second theme was prognosis. It involved algorithms for tools that may help predict the severity of COVID-19 infections and the prognosis and risk of intensive care unit (ICU) admission through the analysis of chest x-ray images. The third theme was long COVID. It involved algorithms for tools that may predict the likelihood of long-term implications (long COVID) resulting from COVID-19 through patient-reported outcomes.

Two focus groups were conducted with 7 physicians to identify which of the 3 use cases were suitable for use in clinical decision support. Participants represented a broad range of medical specialties and had experience providing COVID-19–related care in a variety of venues (Textbox 1).

**Physician representation in focus groups.**

**Medical subspecialties**

- Emergency medicine
- Intensive care
- Palliative care
- Cardiology
- Family medicine
- Diagnostic medicine
- Internal medicine

**COVID-19 care venues**

- Long-term care facilities
- Family medicine centers
- Emergency departments
- Intensive care units
- COVID-19 acute care wards

For each category of possible tools (screening, prognosis, and long COVID), we used the following interview guide questions to seed the discussions: (1) How might a clinical decision support tool focused on *(insert tool type)* be useful in the context of our health care sites? (additional prompts: Could you describe what you see as the value of this type of tool for clinicians [doctors, nurses, and others]? Could you describe what you see as the value of this type of tool for patients?); (2) If we assume that we can access the required data to make the tool work, what additional challenges might a tool like this be associated with? (additional prompts: Any specialized additional clinical knowledge needed? Would it require dramatic changes to existing care protocols or workflows? Any special characteristic of our patient population?); and (3) What types of information or data points would be most crucial in an explanation of the prediction being discussed?

All data from the focus groups were transcribed and loaded into NVivo software (QSR International) for thematic coding.

In parallel, a scoping review of the use of XML for decision support in health care was conducted, using the methods proposed by Levac et al [16]. We generally found very few studies that described testing or methods to collect end-user perceptions of explainability, and even fewer studies that referenced any design theory or framework in the development of decision support tools.

### Define Phase

The objective of the define phase was to synthesize the findings from the work done in the empathize phase and formally define the scope of the problem. For focus group data, we applied a framework developed to study the nonadoption, abandonment, scale-up, spread, and sustainability of technology in health care (NASSS), to analyze physician feedback on the possible tools for development. The NASSS framework is composed of the following 7 domains: condition, technology, value proposition, adopters, organizations, wider system, and embedding over time [15]. The NASSS framework, while traditionally used to analyze technology implementations, can be used to “generate a rich and situated narrative of the multiple influences on a complex project” [17] and assess in advance whether certain technology will be adopted in a health care setting. According to Fereday and Muir-Cochrane [18], a hybrid deductive or inductive thematic analysis was used. An initial deductive coding framework based a priori on the domains of the NASSS technology implementation framework was completed by one of the researchers. During the coding, 2 members of the research team met frequently to review challenges in the coding process and identify new subcodes for each domain. A detailed summary of the NASSS framework domains and sample coded participant data are provided in Multimedia Appendix 1. The objective of this phase was to better understand the suitability of each tool for adoption within the organization. We present a brief summary of the analysis in Table 2.

Physicians felt that the characteristics of long COVID were still unclear and difficult to define, and thus, it would be inappropriate to develop a long COVID tool. There was strong disapproval for a screening tool that makes predictions based on analysis of free text in the patient medical records given the possibility of incorrect information, inconsistent completion of records, missing information, and false reporting by patients.

Physicians were more receptive to the prognosis tool. Their familiarity with x-ray images and more trust in the image data source increased support for this tool. Physicians not only considered this a more useful application but also considered the warning of the impending prognosis to be important.

**Table 2:** Grading of each proposed decision support tool based on the nonadoption, abandonment, scale-up, spread, and sustainability of technology in health care (NASSS) framework domains.

NASSS^a^ domain	Description	Screening tool	Prognosis tool	Long COVID tool
Condition domain	Is the nature of the condition or illness (eg, symptoms, diagnosis, and therapeutics) relevant to the organization?	+^b^	+	−^c^
Technology domain	Can the technology of the tool (eg, underlying algorithm) be supported by quality data sources?	−	+	−
Value proposition domain	Is there potential of the tool to provide some type of business or health system value?	+	+	+/−
Adopters domain	Could the use of the tool result in a change in practice of care providers, and impact patients and their careers?	+	+	+/−
Organizations domain	Are there considerations related to readiness of the institution to innovate, use, and fund new technologies?	+/−	+	+
Wider system domain	Are there political, regulatory, or sociocultural considerations impacting the implementation of the tool?	−	+	+/−
Embedding and adaptation over time domain	Is there potential for the organization to adapt and evolve the tool over time?	−	+	−

^a^NASSS: nonadoption, abandonment, scale-up, spread, and sustainability of technology in health care.

^b^Positive sentiment from physicians.

^c^Negative sentiment from physicians.

### Ideate Phase

In this phase, the research team met continuously to build various approaches for both the user experience (UX) implementation and underlying explainability approaches used in the tool. As discussed in the Introduction section, increasing clinician trust in ML-based CDSS applications is seen as a key driver to increasing their use in actual practice. In line with this principle, the team adopted an evaluation framework published by Tonekaboni et al [4], which presents a series of metrics that can help assess clinician trust in an explanation provided by a ML CDSS tool. The framework includes 3 metrics. The first metric is domain appropriate representation, which represents the degree to which the tool provides adequate information to the end user within the context of the specific clinical setting and workflow. The second metric is potential actionability, which represents the degree to which the tool facilitates the taking of appropriate decisions or “next steps” in the care of the patient. The third metric is consistency, which represents the degree to which changes in the tool’s predictions and corresponding explanations can be explored to determine consistency.

These metrics were incorporated as design guidelines in the ideate phase and then used as the primary metrics in the prototype and test phases.

### Prototype Phase

The objective of the prototype phase was to develop low-cost physical representations of the tool that allow for more detailed end-user feedback and more opportunities to iterate on the design of the solution.

For domain appropriate representation, we focused on providing a succinct summary of additional COVID-19–relevant information that physicians would likely find relevant in the context of a prognostic prediction based solely on chest x-ray images. This included the addition of a subset of patient vitals, laboratory values, and history, including symptom onset.

For potential actionability, we assumed the most important visual component of the tool would be the chest x-ray and corresponding explainability features. The team chose to implement a heat map–based visualization approach that would highlight areas of the image that most significantly contributed to the x-ray severity score. The technical implementation is explained in more detail below.

Our assumption was that this would quickly provide clinicians with the information they needed to make appropriate decisions concerning the next steps of the patient’s care trajectory. In order to provide context to the severity score, we considered a model of ICU admission to assess risk by defining 3 categories of risk (low, medium, and high) and the associated likelihood of admission.

Finally, for consistency, we planned for the clinician to be able to click on multiple imaging results in the patient timeline such that the clinician can compare the ML predictions across images and make assessments as to the consistency of the predictions.

**Figure 1:**
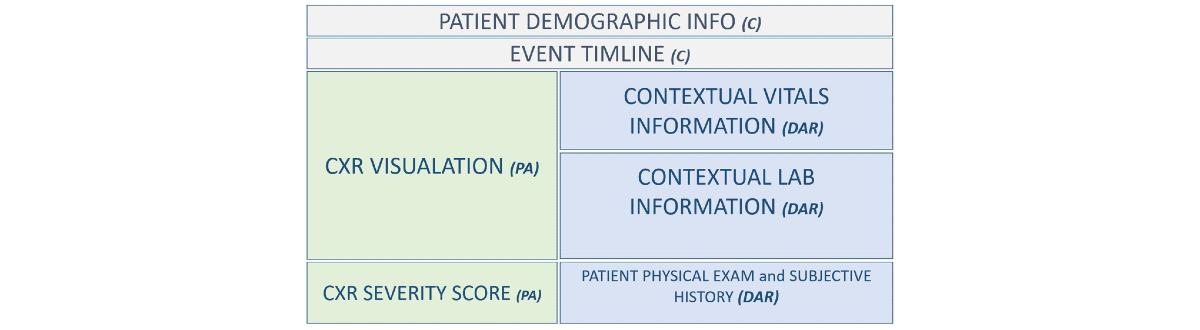
Wireframe prototype indicating different domain considerations. C: consistency; CXR: chest x-ray; DAR: domain appropriate representation; PA: potential actionability.

The prototype wireframe in Figure 1 illustrates the basic design and different domain considerations.

#### ML Model: Developing Explainability

Based on the selected application, we chose to use the algorithm of Cohen et al [19] as the algorithm to include in the prototype application. Their work predicts the level of lung opacity and the geographic extent of disease regions from the input x-ray data, using a deep neural network. The main network consists of a network pretrained on large public non–COVID-19 data sets followed by 2 regression networks, one for each of the opacity and extent outputs. We computed total disease severity as the sum of the 2 neural network outputs. Training of regression networks was performed using COVID-19 data obtained by radiologist scoring of chest x-ray images with the following scores: (1) the extent of involvement of ground-glass opacity for each lung (for a total between 0 and 8), and (2) the degree of opacity for each lung (for a total between 0 and 6).

For our purposes, we were able to leverage their latest publicly available implementation and database [20], thus allowing us to only focus on developing the explainability methods. The multisite data set consisted of posteroanterior deidentified chest x-ray images of patients with varying COVID-19 severity, and each x-ray image had an associated disease severity score obtained by radiologists. Many of the patients had x-ray images from several time points, and the number of time points per patient was not consistent across patients.

We aimed to explore local post-hoc explainability approaches as we needed to explain specific instances of pre-existing models. We focused on model-agnostic methods as they would be useful in broader contexts and be applicable independent of the clinicians’ choice. In particular, we selected LIME (local interpretable model-agnostic explanations) [21] in order to interpret the model output owing to the simplicity in its implementation and perceived intuitiveness of the results. In our case, LIME explained the predicted severity as a linear function of the contribution, positive or negative, that each area in the x-ray has for changing the total severity score. We found the kind of output produced by LIME, which consists of contiguous regions, and this is more consistent with users’ expectations.

#### Low Fidelity Prototype

**Figure 2:**
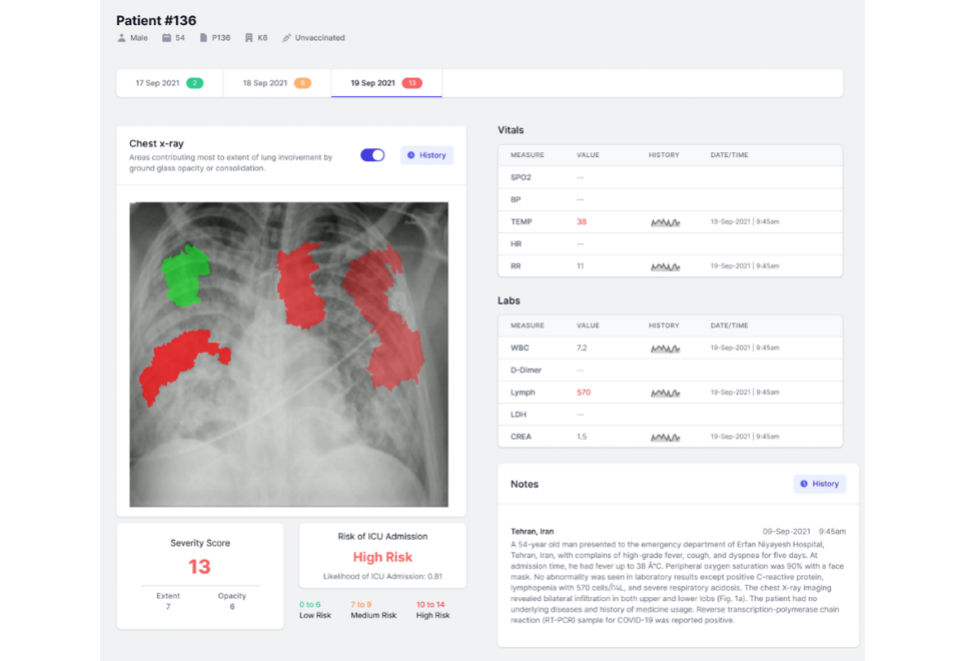
Static low fidelity prototype.

Using our wireframe and the results from the ML model, a static low fidelity prototype of the tool was developed using FIGMA (Figure 2) [22].

We defined 3 levels of the risk of admission to the ICU considering the severity score (low, medium, and high), using the observed ICU admission rates and associated severity scores in our test data set. We defined severity score ranges for each of the risk levels in such a way that similarity in severity was maximized within each range. The risk was then calculated as the average probability of admission within each category. Our data set contained 950 x-ray images representing 472 unique patients. A subset of 398 x-ray images for 162 unique patients with information on ICU stay was used to calculate the risk of admission.

For the explainability component, we used the LIME Python package [23] to explain the predicted severity as a linear function of the contributions from image patches that compose the full x-ray image. Each image was subdivided into patches using the Quickshift Segmentation algorithm [24]. We found that the resulting explanation was unintuitive upon visual inspection, as patches indicating high contributions to increasing severity did not appear to be diseased. In addition, the low fidelity prototype displayed importance values for all patches and proved too confusing during iterative testing. The patches showing a negative contribution were cluttering the display and were unnecessary to show highly diseased regions. This was addressed in future iterations.

According to the design thinking approach [25], we met with 2 clinicians who had participated in the focus group sessions, and asked them to provide individual feedback during informal sessions of 30 to 60 minutes. We asked for their overall impression of the tool prototype design, as well as comments on specific design choices made by the research team to improve UX and explainability. These sessions were conducted over Microsoft Teams. Follow-up was conducted via email as the team made iterations on the comments and ideas from physicians.

### Test Phase

The objective of the test phase was to gain more insight into the working prototype. The high fidelity (software) prototype was developed in Python using the Plotly Dash framework [26], a library that allows for quick prototyping of user interfaces.

The interactive prototype was tested with 5 physicians from the initial focus group sessions. According to Doshi-Velez Kim [27], we used an application grounded evaluation approach in which intended end users used the tool simulating prognostic prediction in the context of a 1-hour semistructured interview. We began the sessions by asking clinicians to explore the application with no guidance and then facilitated a discussion about the various features of the tool by presenting 3 separate patient cases. The questions from the interview guide were as follows: (1) What is your overall impression of what the tool is presenting to you? (overall impression); (2) How well does the tool provide with you with necessary contextual information about the case? (domain representation; additional probes: Is there too much information or is there missing information? Is the information poorly organized or is the presentation of the information confusing?); (3) How does the tool help or hinder your ability to make a treatment decision or take action with the patient? (actionability; additional probes: Is the prediction the tool is making clear? Is the tool adequately transparent with regard to the certainty of the prediction? Are there any complimentary data points you feel are missing for you to make a decision? Do you feel this tool could be shared with a patient in its current state?); and (4) As we show different cases, what are your impressions of how changes in the prediction are explained by the tool? (consistency; additional probes: In cases where the tool shows the progression of predictions for a single patient, does the tool adequately explain the changes? In cases where the tool shows a range of different patients, are the differences in predictions adequately explained by the tool?).

## Results

### Prototype Phase (Low Fidelity)

In evaluating domain appropriate representation, the major information elements required by physicians were present in the first prototype; however, physicians did mention that the following elements needed to be added: (1) A more prevalent display of the date of COVID-19 diagnosis and date of symptom onset; (2) A more prevalent display for vaccination dates if available; (3) The method and volume of current oxygen in L/min; and (4) Additional laboratory test values for procalcitonin, C-reactive protein, and interleukin-6 (indicators of infection or inflammation that could be treated).

As mentioned above in the ideate phase, our objective with this design was to present the prediction in the most significant quadrant of the screen to ensure potential actionability; however, both our clinician testers commented on how presenting the image first violated the basic approach for clinical assessment and decision-making. While we assumed that the additional information displayed in the interface meant to provide patient content would only be looked at if needed, clinicians told us that this would be relevant and would need to be incorporated into their assessment of the quality of the ML prediction. Based on this feedback, we chose to reverse the orientation of the information for the development of the interactive prototype.

In addressing the consistency metric, while we explained to the physicians that the interactive tool would ultimately allow them to move through multiple images to compare the progression of the disease, we could not simulate this in the static image. Physicians did comment that it would be much easier if the multiple images could be displayed in comparison. They also commented on the importance of being able to turn the heat map overlay on and off so as to be able to compare the areas highlighted by the ML model with the actually affected areas on the x-ray image. This suggestion was implemented in the interactive prototype.

Finally, physicians commented that it was unclear how the consolidation in the image contributed to the severity score and how the severity score contributed to the risk of ICU admission. These were addressed in the interactive prototype through the creation of tool tip pop-ups with textual explanations.

### Testing Phase (High Fidelity)

**Figure 3:**
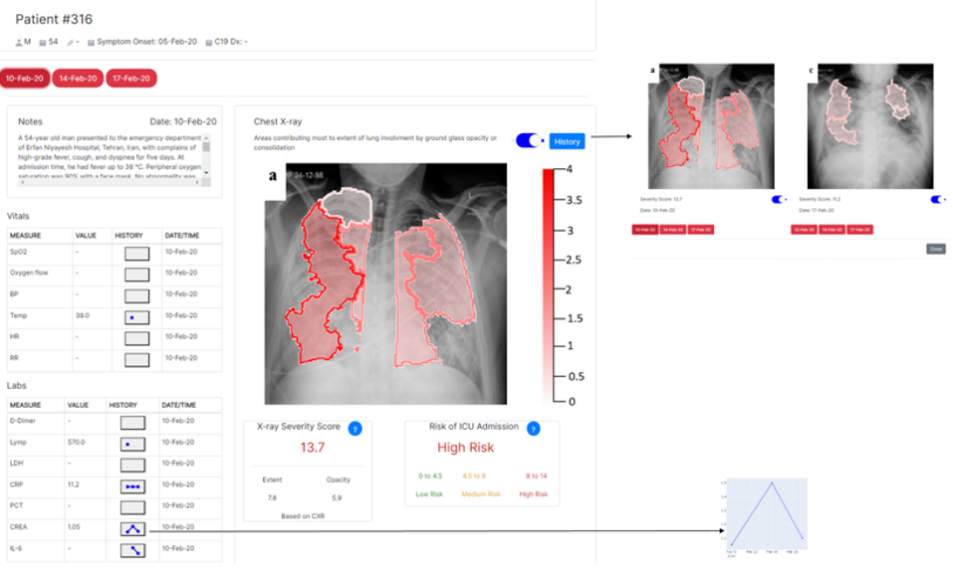
Working prototype.

The final interactive prototype was designed based on all feedback identified from the prototyping sessions and is presented in Figure 3.

#### Domain Appropriate Representation

Clinicians found the design of the prototype elegant, and domain appropriate representation of data was displayed in the tool. The added contextual data (history, vitals, and laboratory values) were deemed necessary, and no items were considered superfluous. Clinicians appreciated that the domain of interest was succinctly represented on a single screen. However, one of the most important issues uncovered was the degree to which physicians erroneously assumed that the additional data present in the tool, namely vitals and laboratory values, were being included in the x-ray severity score. While the tool tips made it explicitly clear that no data other than chest x-ray data were being used in the prediction model, further work needed to be done to visually distinguish information elements used to provide additional domain context from information used in the ML model.

Based on initial feedback during the prototype phase, we specifically probed clinicians on the reorganization of the structure and sequencing of the information in the interface, and the degree to which it supported the standard thought processes clinicians would follow to reach a decision. Physicians responded well to the revised structure and information flow. Moreover, they highlighted the possibility of using the tool as an additional teaching resource with junior staff or nonradiology specialists.

#### Potential Actionability

In order to reduce the clutter in the display of explainability compared to the low fidelity version, we only showed the patches that had a positive contribution to severity in red, where higher values had a darker color. In order to allow the end user to focus on the most predictive areas only, we tested several methods for displaying only a subset of the regions, including displaying a fixed number of patches (eg, 3, 4, and 5) or displaying patches that explained a certain percentage of the final score (eg, 80% and 90%). The selected prototype only displayed the most predictive patches that cumulatively explained 90% of the final score. The rationale behind it is that in some cases where the top contribution comes from many patches with low and equivalent values, by only showing a fixed number of patches, we will be omitting several equivalently important patches. The remaining explainable model hyperparameters (maximum distance, color space and image space proximity ratio, and kernel size for the Quickshift Segmentation; regularization coefficient for the explainer’s ridge regression; and width of the explainer’s exponential kernel) were then optimized by maximizing the coefficient of determination via sequential optimization using decision trees [28]. This optimization process resulted in more intuitive and actionable explanations.

We further explored potential actionability through the following three possible use case scenarios for the tool in the interviews: (1) Triage of patients presenting at the emergency department; (2) Discharge planning; and (3) Shared decision-making with patients.

In the first scenario (triage), many physicians were quick to point out the evolution of care protocols across the different waves of the pandemic. In the first and second waves, the lack of global disease knowledge caused a large number of pre-emptive ICU admissions; however, this is no longer the case. Physicians did note that they felt the tool could be very helpful in terms of planning for potential ICU admissions over time and helping manage staff resource issues.

In the second scenario (discharge planning), we proposed that the tool could be used as a final check for moving patients from high acuity care to lower levels of acuity, either discharge to home or discharge to virtual hospital care. Physicians generally felt that the tool could be useful as an additional data source to confirm an assessment of low risk.

In the third scenario (shared decision-making), we asked physicians whether they thought the tool could be helpful in shared decision-making, especially in scenarios where there may be some disagreement between a physician and a patient about a proposed next step. Physicians noted that the tool could be useful in explaining escalations in care to patients currently experiencing moderate symptoms.

#### Consistency

In terms of consistency, physicians unanimously appreciated the capacity to compare multiple x-ray images in the same view. They also appreciated the ability to toggle the explainability overlay so that both options made it easier for them to assess how consistently the tool was identifying elements of the x-ray image they felt would contribute to overall disease severity. Not all physicians agreed with the tool’s assessments, but felt that more exposure to a larger number of predictions would be necessary for them to gauge how much they trusted the tool.

## Discussion

### Designing for Trust and Decision-Making

As discussed by Wang et al [29], when predictive AI is used in decision support tools, end users seek explanations to help improve their decision-making, and in cases where the tool performs in unexpected ways, explanations are critical for allowing users to identify what elements of the underlying model may be contributing to an unexpected prediction. In our case, we used saliency heat maps to show causal attribution to a severity score, where the highlighted regions represented areas with the greatest contribution to the severity score. Physicians appreciated the ability to toggle the heat map on and off to clearly identify the areas of the image that most contributed. However, multiple physicians did note that trust in the application would be built over longer term use, allowing them to assess the degree to which the application would align or deviate from their own unaided clinical assessments.

As described by Wang et al [29], this may be considered a type of heuristic representative bias, whereby past experience can lead a physician to wrongly associate a current case with similar previous cases. While our design allowed physicians to compare multiple instances of chest x-ray images for a single patient, a further iteration could incorporate features that would help to address this heuristic bias. Specifically, we could include the potential to compare an existing case to similar prototype example cases and use a dissimilarity metric to compare cases.

It is also important to note that there was skepticism that a model based solely on chest x-ray images could provide prediction as good as a model based on multiple inputs. This highlights the design challenge of optimizing domain appropriate representation and potential actionability in the user interface. Clinicians felt that it is important to see the prediction of chest x-ray images in the context of additional clinical information that feeds into their heuristic framework used for assessing a patient’s disease trajectory. Those additional data points clearly played a role in their likelihood to trust the explanation; however, they were not accounted for in our model. Further exploration of this challenge might include comparing the accuracy of the current model to predictions that use additional key inputs.

### Design Thinking and Rapid Evolution

The COVID-19 pandemic evolved rapidly, and as such, the constant engagement with end users allowed the team to improve the potential application of the tool as well as the information displayed. During the prototype phase of development, physicians pointed out that the hospital was rapidly starting up a virtual care service for early discharge of COVID-19 patients to be cared for at home. This allowed the team to realign some of the discussion in the testing phase to assess the suitability of the tool for a unique case that did not exist in the ideate phase of development.

Moreover, it allowed us to modify the scope of information displayed in the tool to bring vaccine-related information into the main display. Again, this information only began to become available in the prototype phase of the project.

Finally, we were able to probe physicians around the applicability of the prototype as a shared decision-making tool to be used with patients, which was suggested informally during the ideate and prototype phases.

The above examples illustrate the importance of agility that is integral to the design thinking approach and represent ways in which the potential applicability and design were improved, which would not have been addressed in a traditional waterfall development approach.

### Combining Design Theory With Additional Frameworks for a More Robust Approach

While design theory provides a well-established approach for continuous engagement with end users, we believe our approach of augmenting design thinking by incorporating additional conceptual frameworks helped to create a more robust collaborative tool design.

First, we used the NASSS framework during the “define” phase of the project to systematically analyze the results of our physician focus groups. This approach helped the team to quickly identify how the potential solutions would align with the various subdomains of the model. We see this as a pragmatic approach and helpful augmentation of the design thinking process to ensure the chosen design direction does not face dramatic sociotechnical barriers to development and potential implementation. Recent research into adoption of ML into clinical practice has used the NASSS framework in a similar manner. Pumplun et al [30] used the NASSS framework to identify 13 specific factors influencing the adoption of ML systems and further proposed a maturity model to be used by health care institutions to assess their readiness to adopt ML-based tools.

Similarly, we augmented the ideate, prototype, and test phases of the project by applying the evaluation metrics proposed by Tonekaoni et al [4]. This approach allowed us to begin the ideation and design process focused on core domains that would impact physician trust (domain appropriate representation, potential actionability, and consistency). It provided a consistent lens to assess both the prototype and test versions of the tool. This consistency in approach led the team to quickly identify design-specific improvements that directly led to the production of a prototype our physicians felt could provide immediate value.

Overall, the approach taken in our work can be situated in an evolving space concerned with incorporating AI technologies into health care software. Anderson et al [31] proposed a framework of 5 lenses from which to view this growing research field. Our work makes contributions to 2 of these lenses (AI as alignment with human values and AI as a design process). First, we addressed the alignment of AI as it relates to clinician trust, describing an approach to wire framing and prototyping that incorporates the use of a theoretical framework for trust in the design process itself. We described how this allows to gauge end-user alignment or trust in AI at multiple stages and optimize designs accordingly.

Second, as described in detail throughout this work, we propose that the alignment of AI is dependent upon integration of end users throughout the larger design process. Our work shows the importance and value of engaging end users prior to tool development, specifically in the process of assessing the broader applicability of a potential AI tool and its eventual use within actual health care environments.

### Limitations

There are several limitations associated with this work. First, from a ML perspective, though we can verify the intuitiveness of the explanation, the accuracy of explainability methods has not been properly studied to date. We thus do not know how well an explanation fits the true underlying prediction in spite of its level of intuitiveness. This is of particular concern in the case of additive feature attribution methods like LIME, where a local linear model is used to explain a potentially more complex nonlinear underlying model.

Second, we used a publicly available data set with limited data, and thus, there were several implications. For example, it was difficult to find exemplary samples where an explanation can clearly demonstrate why an algorithm deviated from the ground truth or examples that can shed light on why an algorithm may have behaved in an unpredictable way. The data set contained a lot of missing data and was limited beyond imaging information, and as such, it was challenging to find examples with the full patient state (such as vital signs, multiple time points, etc) to provide end users with the desired contextual information to make a fully informed assessment. Finally, the data set was relatively old considering the rapid evolution of COVID-19 and approaches to its treatment, and this has implications on the likelihood of ICU admission considering the state of a patient.

With regard to our focus group participants, it is important to note that only physicians were represented in this research. While this was intentional in the study design, as it is primarily physicians who will make decisions about ICU admission, our work could have benefited from the inclusion of additional health care providers, such as nurses and respiratory therapists. Indeed as Nalin [32] pointed out, a larger system perspective of the use of the proposed tool could have provided richer data in the focus group phases.

### Conclusion

Our work set out to use a design thinking approach to develop an XML-based decision support tool to assist clinicians. We augmented the design thinking approach by using the NASSS framework to help inform the development focus and direction, and added a formal evaluation framework from the report by Tonekaboni et al [4] to continuously focus our design on elements that would improve clinician trust in the tool. This research contributes to the body of health care literature that deeply integrates end users into the design and evaluation of XML in clinical decision support tools. As discussed, clinician trust is seen to be one of the key barriers to larger scale adoption of ML-based clinical decision support tools.

We believe that the approach described in our work is a unique and valuable contribution that outlines a direction for ML experts, UX designers, and clinician end users on how to collaborate in the creation of trustworthy and usable XML-based clinical decision support tools.

## Appendix

Multimedia Appendix 1 Description of nonadoption, abandonment, scale-up, spread, and sustainability of technology in health care (NASSS) categories and example physician comments.

## Abbreviations

AI: artificial intelligence
CDSS: clinical decision support system
ICU: intensive care unit
LIME: local interpretable model-agnostic explanations
ML: machine learning
NASSS: nonadoption, abandonment, scale-up, spread, and sustainability of technology in health care
UX: user experience
XML: explainable machine learning

## Data Availability

Two separate data sets were used in the completion of this work. The data sets used for developing the underlying disease severity and outcome prediction models are available through the repository of Cohen et al [2,20]. The focus group data generated and analyzed during the study are not publicly available owing to the consent obtained when the data were originally collected.
