# Supplementary material for: Integrating Explainable Machine Learning in Clinical Decision Support Systems: Study Involving a Modified Design Thinking Approach
Source: JMIR Form Res. 2024 Apr 16;8:e50475. doi: 10.2196/50475 (PMC11061789; doi:10.2196/50475)
Supplement: Multimedia Appendix 1 [file formative_v8i1e50475_app1.docx]

**Multimedia Appendix 1.** Description of nonadoption, abandonment, scale-up, spread, and sustainability of technology in health care (NASSS) categories and example physician comments.

(1) The Condition Domain pertains to the nature of the disease and all the parameters surrounding it (symptoms, diagnosis, testing, prevalence, therapeutics etc.) and the degree to which they are understood. While both the Screening and Prognosis tools had well defined characteristics for the Condition Domain, physicians felt the characteristics of Long COVID were still nebulous and difficult to define. As such, only the Screening and Prognosis tools passed this domain.

*“What might be considered a post COVID syndrome, as opposed to COVID itself? For some people it's neurocognitive, for some people it's a cardio, it's carditis or pericarditis, and for other people it's chronic fibrotic lungs and persistent cough. And it could be totally different without overlapping at all. So how would that be defined, ‘long term COVID’? Or is it just that we're saying that the patient has symptoms beyond 28 days of any kind related to COVID?”*

(2) The Technology Domain pertains to the nature of the tool in question, including key features, knowledge it provides end users, knowledge required to use it, etc. As our discussions focused on a proposed XML tool, much of the discussion centered around trust in the reliability/accuracy of the underlying algorithm, and trust in the quality of available data to be used in the tool.

There was strong disapproval for a Screening tool making predictions based on analysis of free text in the patient medical record. This included incorrect, inconsistent completion or missing information in charts/EHR, plus false reporting by patients.

*“Because we don't have electronic healthcare records, we don't have a rigid way of finding that data and I know it's free text, but I'm just worried about the quality of what you're analyzing …. I don't know if you've ever seen a doctor's notes.”*

*“At the clinic, we would have nonstop storytelling only to receive people in the room telling us that they're congested or having a cough with fever etc. and they didn't want to be turned away”*

In regard to the second proposal, the Prognosis tool, physicians were more receptive to this. In addition, there familiarity with x-ray images and more trust in the image data source increased support for this tool

*“I think overall the idea of giving a prognostic rather than diagnostic thing is probably likely to be more acceptable to physicians. If you're telling me the patient’s diagnosis, I'm a lot more likely to distrust and be suspicious, but if you're telling me, oh, I agree, given the diagnosis, how sick the patient is, I think that's more acceptable somehow. You know that doesn't obviate the need for explainability, and to display something to me about the case and why it's true”*

*“We can all look at x- ray and say ‘that doesn't look too bad’, and we could all look at an x- ray and say ‘Oh my God’. So yes, it is nice to have that ‘Oh my God’ translated into something that's a little bit more objective.”*

To improve trust in potential CDSS tool using chest x-rays, some physicians believed other parameters needed to be included (labs, vitals) in the model.

*“Does an x-ray itself give us the information that we want to get? And if you're making a model to look at prognostication, my suggestion would be to make it broader than looking at one piece of information.”*

*“Maybe the x-ray is enough precision in a digitized manner, maybe the patterns are sufficient, but I have to believe that incorporating more data points such as blood values and inflammatory markers would be even more beneficial and add something extra on top of it.”*

Physicians with more digital experience expressed confidence in this type of prototype.

*“In cardiology and radiology, there's a fair amount of experience with machine learning in imaging analysis because it's all digital and the whole concept of machine learning is it doesn't have to just give the gestalt, it can go down pixel by pixel over the entire frame and decide and pick up patterns and patterns once they've been correlated with clinical outcomes, can be very predictive.”*

As a results of physician discomfort around the ill-defined concept of Long COVID, physicians were reluctant to trust that an algorithm could discriminate between a potential Long COVID symptom and pre-existing underlying symptom.

*“Long term COVID is certainly a nebulous entity still now. So, defining the endpoint which could itself have multiple different definitions, but overlap with other conditions that may not be COVID at all would make it a tricky thing to tease out. I'm just saying it might not be as clean as the other ones could be.”*

(3) The Value Proposition Domain focuses on specific scenarios where a tool could be seen to bring some type of business or health system value. For a Screening tool, physicians commented that during active waves of the pandemic, the level of attention paid to COVID-19 symptoms is very high, and the protocol for the emergency department was to test all patients as part of admission, which made the tool somewhat redundant. However, there was mention that a screening tool might be useful as an early warning system as cases decreased and less specific attention is paid to COVID-19 related symptoms.

For the Prognosis tools, physicians not only considered this a more useful application but considered the warning of impending prognosis to be important.

*“I think it's more applicable because we don't have a test that we can get very quickly that gives us an answer. It is a clinical prediction rather than an objective prediction. I definitely see this as much more useful. Same thing for long term outcomes. Is this person likely to have functional disability? Is this person likely to have a pulmonary disability? Does this person need to be monitored more closely when they leave the hospital? Do they need some sort of rehabilitation program? Do they need psychological counseling? All these kinds of things like outcomes, I definitely see this tool being used for these kinds of predictions.”*

They added this prototype would be helpful to manage health system resources, especially during outbreaks in long term care facilities, and sub-acute hospitals to avoid unnecessary transfers to the ER, help to gauge the potential impact on the ICU during heightened admissions in COVID-19 waves, as well as some care based shared decision making scenarios with patients.

For the Long COVID tool, physicians felt that the low volume of patients with Long COVID symptoms, combined with the lack of straight forward treatment options contributed to a potentially lower value tool.

(4) The Adopters Domain focuses on potential changes in roles and practice of care providers, as well as any additional responsibilities or impacts on patients, relating to the use of a tool. The Screening tool was discussed in the context of family medicine and the ER. For family medicine, the tool would have little impact on care providers or patients as the tool would screen and identify patients at risk of COVID-19 which is part of the current telemedicine practice. However, physicians did comment on the need for EHR driven structured data capture to facilitate the accuracy of the tool.

“They (family practice clinic) have an electronic health care record that is reasonably rigid. I'm sure by now they've actually put in things like: Do you have the vaccine? I'm sure by now they have multiple results, not just free text, but you actually have lab results there too. And you know I'm sure they have x-rays. I would go for something like that.”

As mentioned earlier, during active COVID-19, the protocol of the ER was to test all patients for COVID-19 so the tool would simply be redundant.

For the Prognosis tool, our physicians suggested an array of medical subspecialties could adopt this prototype including ER physicians, GPs, respirologists, and any physician who needs to determine whether a patient must be transferred to the ICU. Regarding cardiologists and radiologists, it was mentioned because they have more experience in digital imaging analysis, that they would likely be more willing to adopt this prototype. Some are aware of ML techniques and thus are comfortable using this.

With regards to the Long COVID tool, physicians considered the possibility it could be adopted by GPs in outpatient settings but not in medical sub-speciality outpatient clinics since there are multiple vague symptoms affecting several areas of the body and as such better a general practitioner address it.

“A gastroenterologist at the very beginning ...was going wild because all these people have abnormal liver function tests, so he was actually booking them all into his clinic and then he was saying, ‘Oh my God, what am I going to do with all these different people?’ So, it would have been reasonable to say ‘no’, you shouldn't be doing a follow up, your GP should look after three months.”

(5) The Organization(s) Domain which includes the readiness of the institution to innovate or use new technologies was generally positive for all suggested prototypes. For the Screening prototype, the organization where the physicians work has seen innovations in telemedicine, has many specialized outpatient clinics, and has a primary care unit with an advanced EHR and promotes training of medical students and residents. This favours the use of this prototype in this organization; however, physicians were quick to note that because of the poor record keeping, lack of EHRs in all departments, the screening tool would fail. Alternatively, the Prognosis tool could succeed in this domain because the organization can not only make chest x-rays available but also provide access to laboratory data and additional objective information for use in the tool. The organization is also at the forefront to create a fully virtual hospital with remote monitoring and care for patients outside the physical hospital. As such, specialized support tools would be seen as part of movement to embrace digital tools to provide better patient care. For the Long COVID prototype, the organization had set up a Long COVID outpatient clinic, and while the tool could be used in this scenario, the clinic was still in its formative stage during the time period of the focus group sessions.

(6) The Wider System Domain is concerned with the degree to which larger political, economic, regulatory considerations, external to the organization, might impact the implementation of a tool. In this domain, all research into machine learning tools face similar challenges around the Internal Review Board approval for use of the large amounts of patient data needed to facilitate testing and prototyping. Indeed, our research was completed using publicly available data sets for this reason.

In the context of the Screening prototype, physicians indicated additional data would be needed to better operationalize the potential risk a patient poses, for example, employment data, vaccine status , etc. While it is possible that this data could be collected by administrative health care professionals, access to broader provincial data sets was seen as a challenge to making a Screening tool useful.

*“Was there an outbreak at a school? … I would want to know this…Do they have a caregiver? And has the caregiver been vaccinated? Or were they working in three or four different places? What worries me is they're totally different analysis. So, it's not just nine (factors) that pertain to the person …So that would be my problem, not just nine. It would possibly be like 40.”*

(7) Finally, the Embedding Adaptation Over Time Domain, is concerned with the organization to adapt and evolve the tool over time. In this domain, only the Prognosis tool was seen as suitable as physicians discussed the possibility of evolving the underlying model over time to include additional data inputs beyond chest x-rays as well as the possibility of developing protocols to support serial x-rays for use in the tool.
